# Public attitudes towards sharing loyalty card data for academic health research: a qualitative study

**DOI:** 10.1186/s12910-022-00795-8

**Published:** 2022-06-07

**Authors:** Elizabeth H. Dolan, Kate Shiells, James Goulding, Anya Skatova

**Affiliations:** 1 N/LAB, Nottingham University Business School, University of Nottingham, Si Yuan Building, Jubilee Campus, Nottingham, NG8 1BB UK; 2 Medical Research Council (MRC) Integrative Epidemiology Unit, University of Bristol, Bristol, UK; 3 Department of Population Health Sciences, Bristol Medical School, University of Bristol, Bristol, UK; 4 Alan Turing Institute, London, UK

**Keywords:** Attitudes, Cancer research, COVID-19 research, Health research, Loyalty card, Personal data, Qualitative, Safeguards

## Abstract

**Background:**

A growing number of studies show the potential of loyalty card data for use in health research. However, research into public perceptions of using this data is limited. This study aimed to investigate public attitudes towards donating loyalty card data for academic health research, and the safeguards the public would want to see implemented. The way in which participant attitudes varied according to whether loyalty card data would be used for either cancer or COVID-19 research was also examined.

**Methods:**

Participants (N = 40) were recruited via Prolific Academic to take part in semi-structured telephone interviews, with questions focused on data sharing related to either COVID-19 or ovarian/bowel cancer as the proposed health condition to be researched. Content analysis was used to identify sub-themes corresponding to the two a priori themes, attitudes and safeguards.

**Results:**

Participant attitudes were found to fall into two categories, either rational or emotional. Under rational*,* most participants were in favour of sharing loyalty card data. Support of health research was seen as an important reason to donate such data, with loyalty card logs being considered as already within the public domain. With increased understanding of research purpose, participants expressed higher willingness to donate data. Within the emotional category, participants shared fears about revealing location information and of third parties obtaining their data. With regards to safeguards, participants described the importance of anonymisation and the level of data detail; the control, convenience and choice they desired in sharing data; and the need for transparency and data security. The change in hypothetical purpose of the data sharing, from Covid-19 to cancer research, had no impact on participants’ decision to donate, although did affect their understanding of how loyalty card data could be used.

**Conclusions:**

Based on interviews with the public, this study contributes recommendations for those researchers and the wider policy community seeking to obtain loyalty card data for health research. Whilst participants were largely in favour of donating loyalty card data for academic health research, information, choice and appropriate safeguards are all exposed as prerequisites upon which decisions are made.

**Supplementary Information:**

The online version contains supplementary material available at 10.1186/s12910-022-00795-8.

## Background

As digital technologies become more ubiquitous in our lives, the personal data they collect are being increasingly utilised to assess a range of factors, from health to wellbeing [1]. Recent reports [1, 2] recognise this development and discuss how the definition of ‘health data’ may need to diversify, with individuals’ health now being inferable from digital data produced via technologies such as wearables, smartphone apps, as well as data sources such as shopping data or phone records. Reports highlight the importance of individual agency in this area, and question what opportunities there are to aid people in participating and influencing the structures that govern health and data. This study acknowledges the value of involving the public in the development of an ethical framework for donating loyalty card data for research and examines its acceptability and the importance that the public attaches to individual agency when sharing this form of data.

Loyalty cards were widely introduced by stores as both a marketing tool and a means for retailers to encourage customer fidelity by rewarding an individual with benefits [3]. Loyalty card data now represents one of the oldest types of personal digital data, capturing information on the food and products purchased nationwide for over a quarter of a century. Information collected includes item type, spending category, item price, timestamp, and store location. Currently, there is very limited research on using loyalty card data to assess individual and population health. However, a selection of studies shows the potential of loyalty card data for health research [4–11]. Davies et al. [5] used loyalty card data to show a positive correlation between changes in air quality and purchases of cough and cold medication, hay-fever, and pain relief using data of 10 million customers from a major UK supermarket. Dzogang et al. [6] leveraged loyalty card data to evidence the relationship between over-the-counter medication sales and seasonal indicators of mental health across the UK. Using loyalty cards data from 1.6 million customers combined with GP open practice prescription data, Aiello et al. [7] found positive correlations between both calorie consumption and consumption of carbohydrates, fat and sugar with hypertension, cholesterol and diabetes, whilst nutrient diversity was associated with lower prevalence of disease. These studies aggregated customer transactions to geographical areas, but did not take account of purchasing patterns at the level of individual customers, risking 'ecological fallacies' being inferred when results are interpreted. In contrast, Jenneson et al.’s recent study of data from 50,000 loyalty cards [8] linked transactions to pseudonymised customer IDs. On investigating purchasing patterns of fruit and vegetables for individual customers, they discovered associations between lower purchasing of fruit and vegetables and lower age ranges, affluence, and monthly spending. All of these studies emphasise the potential for loyalty card data to offer new information on population purchasing and its links to population health. However, the limits of such research are often dependent on how such loyalty card data are accessed, the level of detail shared and the types of health data that can be linked to loyalty cards, either on a regional or individual basis.

Public acceptability is also key to repurposing loyalty card data for health research. Previous studies have demonstrated public antipathy towards health data and partnerships with commercial companies [12–15]. However, giving commercial data to not-for-profit organisations to conduct health research reverses the direction of data flow often seen in traditional private and public sector healthcare partnerships. Historically, such partnerships increase private sector access to public sector health data [14]. The opposite is true for the sharing of loyalty card data for health research, where it is the public sector that benefits from access gains. Yet the opportunity for the public to understand re-purposing of loyalty card data for health research is a more complex one, with the concept relying on an unconventional public–private sector partnership that raises additional red flags in individuals’ perception of such linkages—even though data is moving from private to public sector, rather than the other way around.

Studies into the public acceptability of sharing data for health research, and further linking this data with health outcomes and other health-relevant data, have established that whether or not data has been used on its own or linked to other datasets, the public expects safeguards to be in place to ensure appropriate data security, and desired levels of anonymisation of their data in order to address fears about privacy for both quantitative and qualitative data [12, 16–19]. Context of the use case and the specific data type is key in determining an individual’s willingness to share personal digital data [12, 20–23]. Previous studies have focused on mobile phone and app data [20–23], emphasising the need to specify the data type being shared when gathering public opinion. For example, location data was often viewed as more sensitive than other types of information [20–23]. Further, Jones et al. [13] explored public attitudes towards using mobile phone call records for health research and found participants had concerns that their data could be misused and even sold to employers or insurers. In terms of research on loyalty card data, Skatova and Goulding [24] showed that over a half of a large survey sample were willing to donate their data for health research. They demonstrated that the likelihood to donate was increased when the intention to donate was motivated by social duty, and by understanding the purpose of donating data.

Further research has examined the public acceptability of linking loyalty card data with health records for research. Tully et al. [12] used survey data across two countries to conclude that caution is needed when linking data from the commercial sector to health, with participants preferring the linkage of health records to private sector records least from all options. They concluded that greater understanding of public preferences is necessitated before this type of data linkage takes place, especially with involvement of the private sector in health already known to be controversial. Clarke et al. [25] asked those who said “no”/”not sure” to the linkage of shopping records to health data about what might change their mind. Natural language processing was used to analyse free text responses, producing a themed analysis based on the number of occurrences of words, phrases, and topics. Nine themes were identified, and from these themes key barriers to sharing loyalty card data isolated, including: data security, privacy, inaccuracy of data, not understanding purpose, and simply not using loyalty cards. Although Clarke's methodology allowed for efficient analysis of large-scale free text response data, it may not have identified all nuances within the natural language responses provided, and captured all intended meanings from participants.

In a qualitative study, Skatova et al. [26] investigated attitudes to direct linkage of loyalty card data into a data bank of a longitudinal study with participants of the Avon Longitudinal Study of Parents and Children through semi-structured focus groups. Similar to previous findings [24, 25] they found that a key factor affecting willingness to share the data was understanding the purpose for which loyalty card data would be used in health research. They observed that attitudes towards donating data evolved throughout the timeline of the focus groups, and as the proposed usage and benefits became clearer, willingness increased commensurate with a desire to cure disease and help society. They also found that the need for safeguards was important in the use of their data, with, participants emphasizing concerns about identification when data of higher granularity is shared. This was accompanied with concern about potential for data misuse, and a desire from participants to maintain both a level of control over their data and assurance that data would be used only for not-for-profit purposes.

While commercial use of the data shared for health research was never assumed or discussed in the study’s focus groups, with the facilitator clearly stating that discussion was limited to use of the data for public good, the narrative of whether shared data might be used for profit remained present in conversations. This highlights the need to understand both fears and expectations the public has about personal data being repurposed for research, and the levels of detail and granularity of shopping history data that are deemed acceptable and appropriate for researchers to access. Public acceptability is also likely to alter according to the proposed health condition under investigation, and their current relevance, with those conditions more current in individuals' minds (e.g., COVID-19) being more likely to be viewed as acceptable research focuses. However, there has been no published investigation into whether people have preferences for different kinds of health research that their loyalty card data would be used for, and whether a project on one or the other health condition can be more enticing for individuals to donate their data. Previous studies do suggest that a difference in the public’s willingness to share other types of digital footprint data is dependent on the health condition being investigated. Franklin et al. [22], from a survey of cancer patients, found that 34% of participants were willing to share mobile application data for health research, whereas willingness to share mobile application data in order to address mental health rose to 68% [20]. In another study, individuals were interviewed both before and after the COVID-19 pandemic started in 2020, finding that 77% of those who were previously not willing to share their digital data for use in public health had changed their mind, additionally stating that they also intended to download a COVID-19 specific contact tracking app [27].

While studies indicate an apparent willingness to donate loyalty card data in general, consent appears dependent on an understanding of the concept of data donation itself, the level of data safeguards in place, and the level of transparency in the direction of data flow, from private to public. The level of safeguards, however, that are expected by the public for loyalty card data for use in health research is still unclear and could change depending on their level of understanding and attitudes towards this research type. Similarly, the type of health conditions to be investigated using personal data could also affect public opinion; differences may occur, for example, when data shared for chronic disease compared to infectious diseases. Therefore, this study had the following three overarching aims—to assess: (1) What are the most widely held public attitudes towards donating loyalty card data for academic public health research? (2) Which safeguards would the public want to see implemented in order to donate their loyalty card data for health research? (3) How do participant attitudes vary according to whether loyalty card data would be used for cancer or COVID-19 research?

## Methods

### Study design

To address these goals, semi-structured qualitative telephone interviews were conducted to gauge public opinion on attitudes to donating loyalty card data for research. Interviews began with a brief icebreaker exercise, where participants were asked to reflect on any sensitivities they attached to personal data and the implications of donating this data for research. Remaining interview questions were framed around donating loyalty card data. Half of participants were asked about donating loyalty card data specifically for COVID-19 research (N = 20) (interviews conducted by author KS), and half for ovarian and bowel cancer research (N = 20) (interviews conducted by author ED). Participants were also asked about their views on donating loyalty card data for health research in general. The interview guide was divided into questions on the following three themes: understanding, control, and trust. These three themes were found to play an important role in previous research with longitudinal cohort participants [26]. The full interview guide can be found in Additional file 1: Appendix S1.

### Participants, recruitment strategy and compensation

Participants were recruited through Prolific Academic [28]. Full information about the study, along with the informed consent form and demographic questions, were delivered via Qualtrics [29]. Participants then registered their availability for the telephone interview. Recruitment was only restricted to those based in the United Kingdom, aged 18 or over. After completing 30 interviews, maximum variation sampling was then used in order to increase the diversity of the sample and the generalisability of the results. As the majority of participants from the first round of recruitment were under 40 years of age, recruitment of the final ten participants was limited to those over 40 years of age. The final sample included 55% Female, 60% under 40 with majority of individuals—60%—having at least an undergraduate degree. Participants 101–120 answered questions in regard to COVID-19, and participants 121–140 answered questions in relation to ovarian and/or bowel cancer (female participants were asked about both types of cancer, and male participants about bowel cancer). The groups were assigned randomly yet remained broadly comparable, with those questioned about COVID-19 having an average age of 33 and being 60% female, and those questioned about cancer having an average age of 36 and being 50% female. Participant characteristics can be found in Additional file 2: Appendix S2, Table S1. The duration of interviews was approximately 20 to 30 min, and interviews were recorded using a voice recorder. After completing the interview, participants were reimbursed £10 via Prolific Academic.

### Data analysis

Interviews were transcribed verbatim using a university-approved transcription service. Transcriptions and analytical notes taken during the interview were imported into NVivo version 11. Directed content analysis [30] was used to sort quotes into two a priori themes relating to the overarching aims of this study: attitudes and safeguards. Researchers then identified sub-themes corresponding to these two a priori themes using the conventional content analysis approach. Authors KS and ED carried out the coding process independently. ED coded all 40 interviews and KS coded 10 percent randomly selected to compare and confirm findings. Both authors concluded that data saturation was met with identified themes recurring throughout the 40 interviews and no additional concepts gathered from later interviews.

## Results

The attitudes and safeguards expressed by participants are summarised thematically below. With regards to differences in attitudes according to the proposed type of health condition for which the data would be used, the change in research topic discussed in the interview from COVID-19 to ovarian cancer and/or bowel cancer, made no difference to the rate of participants happy to donate loyalty card to a data donation bank; an equal number of participants (15 out of 20 in each condition) would be happy to give their data to a databank which would allow researchers to securely leverage that loyalty card data for different types of health research as and when it was required. However, there were differences depending on diseases that were discussed in terms of participants’ understanding, and willingness to donate different data types. These are reported below. There was a small difference in preference for sharing health status alongside loyalty card data, where rates were higher for ovarian and/or bowel cancer compared to COVID-19 condition: 17 out of 20; 15 out of 20, respectively.

Two sub-themes were identified in relation to the a priori theme “Attitudes” and were labelled as (1) rational response, and (2) emotional response. We define rational response as based on the assessment of available information and understanding, while emotional response reflects ‘gut’ feelings and instinctive thoughts. In terms of the a priori theme “Safeguards”, we defined three sub-themes, which were labelled as (1) anonymisation/the level of data detail; (2) control, convenience and choice in sharing data; (3) transparency and data security. The frequency of participant responses can be found in Additional file 3: Appendix 3, Table S2. We discuss each theme in turn below.

### Attitudes

We asked participants in two subtly different ways whether they would donate their data, and their responses were consistent. First, participants were asked if they would donate their shopping data to health research in general, and second if they would donate their shopping data to a databank where researchers would use the data for various specific health research projects. In both cases, a majority of participants (34 out of 40) were happy to donate data. For most participants there were no health research types they would not want their loyalty card data donated to. Participants were informed about loyalty card data types which could be shared: spending category, item price, timestamp, and location. The majority were willing to share all these data types (28 out of 40). The discussions that these responses evoked were both rational and emotive.

#### Rational response

There were three main reasons why participants were willing to donate their loyalty card data. First, participants would donate because they conferred importance to health research, because donating their data was both beneficial and helpful to society. Participants generally wanted to help by donating their data and felt if it was helping society or would “*make people well*”, they would be happy to do so (P123, similar in P116).

A second cluster of reasoning behind donating shopping data was whether they considered shopping data private. Those who did not consider the data private did not mind sharing it. The majority of participants were less concerned about donating loyalty card data than other forms of personal data, often stating they were “not bothered” about loyalty card data being shared and displaying attitudes of indifference: *“I’m happy to share everything and once it’s out there, it’s out there.”* (P102). This lack of concern was attributed by some participants to the fact that this information was public already as they were visibly purchasing in shops, and the retailer already had their loyalty card data. For example, P104 stated that: *“It doesn’t really bother me if people see that, especially if it’s used for research purposes […] I wouldn’t consider it personal. I mean, you’re in a public place anyway.”*

However, there was a small number of participants (N = 2) who both considered shopping data private and gave negative responses to donating the data to medical research. They were sceptical about whether the data could be kept secure if it's being used for research (P112) as well as not understanding how the data could be helpful for research (P103).

Several participants (13 out of 40, 10 of those in the COVID-19 condition) were initially confused over how donating loyalty card data could be useful in medical research: e.g.: “*I think like if someone gets in contact with someone that had COVID maybe[…] oh I’m not sure how they would track it if they don’t have the person’s address or their personal information.” (P110).* In the bowel cancer condition initial understanding was much higher (17 out of 20).

After a short explanation, or participants themselves reflecting on the topic, most individuals developed a sound understanding of the concept of using loyalty cards data for medical research, for example to understand *“the relation between the products being bought, […] and the factors that are observed in COVID-19 patients.” (P112).* Further, the majority of participants in both conditions (24 out of 40) recognised the potential of loyalty card data to investigate disease causation through diet. For example, P137 said that: *“[…]they [researchers] would be categorising a variety of products that would be, I don’t know, a, b, c, d in terms of contributing towards bowel cancer. Um, being able to match those kind of things it would give them trends […] are people […] more likely to be, you know, susceptible to bowel cancer because they’re buying products x, y and z?”.*

This understanding increased throughout the duration of the interviews, and participants began to envisage the concept: *“it’s a great idea […] everybody should be doing it.” (P113).* They talked about methods of collecting these data that can work best for the researchers, and interventions the research could lead to. Gaining an understanding of the potential of shopping data in health research throughout the interview helped most participants to form a positive attitude towards donating these data to medical research. Once the uncertainty of how their loyalty card data would be used in health research had been addressed, participants actively wanted loyalty card data to be used for medical research with the concept seen as beneficial:*“[…]these big datasets which our society has created could – can be a real force of good..” (P140).*

#### Emotional response

Even though the majority of participants would be willing to donate their loyalty card data, fears about data sharing were still disclosed. Some participants would donate but were cautious or reserved about donating, dependent on the circumstances: *“[…]it’s good, […] I’m okay for donating it but I’m not sure how it may be used, […] it depends on who, who the data is passed on, so which organisation is using my data and how it is using it.” (P134).*

Over a third of participants (14 out of 40) were worried about donated loyalty card data being obtained by a third party. Most of these participants feared that it would be used by private companies to market and advertise products to them, even though such a possibility was never mentioned or implied. There was a distrust of the motives of private companies, and a belief that profitability would take precedence over the welfare of participants: *“So, fears I guess would just be that data kind of being breached and that privacy being breached and being used for things that aren’t beneficial […] just for kind of like advertising or – like algorithms that kind of promote certain products to you that are actually detrimental […] people’s data just being like sold – er – to loads of different companies.”(P138).*

There was further sensitivity amongst participants about sharing location data. A number of participants indicated they would not donate data with associated location information (10 out 40). Participants frequently mentioned their “address” when asked which personal data was the most sensitive. Participants were informed that loyalty card location data would not necessarily correspond to their address but rather a store location or geographical region. Nonetheless, location data was often negatively associated with surveillance, with concerns being more identifiable in those participants answering questions about COVID-19. Participants questioned why this data type was needed: *“[…]except location, because, that’s just getting too much […] you can determine exactly my pattern of movement around London […] a little bit much for just health research.” (P117).* Those whose interview included questions about COVID-19, frequently proposed loyalty card data could be used to track individuals’ movements when asked how it could be useful for health research. In response to the same question, in interviews where the specific research topic had been cancer, no participants mentioned using location data in this manner. However, the fact that a participant voiced concern that location data could be used to track movements did not translate to a decreased willingness to share such data; the majority interviewed in the COVID-19 scenario responded positively to donating location data (19 out of 20). In comparison, nearly half of participants (9 out of 20) interviewed about cancer were not prepared to share location.

Despite expressing potential fears, most participants indicated a high level of trust in researchers, although some stated that the level of trust they could confer would be dependent on who the researcher was. Only one participant answered they would not trust researchers at all. Participants often contextualized the higher trust they had in researchers by comparing them to private companies in whom they had less faith: “*I would trust university researchers more than private researchers” (P109).* In particular, they referred to the high standards universities maintained across either ethics, working towards good causes, or data policies.

### Safeguards

While the majority were positive about data donation, even those who were positive mentioned some caveats in the sharing of their data, with the importance of the following items being common: data anonymisation; data security; and a transparent choice as to what their data was used for. We discuss these three issues related to safeguarding the use of loyalty cards data in turn below.

#### Anonymisation and the level of data detail

Participants' statements on their happiness to share loyalty card data often came with a stipulation that their information was kept safe and secure. However, the main caveat given was that the information could not be used to identify them. The main concern of participants was their name being shared, and a higher level of detail of information was associated with a higher chance of being identified. These concerns fit with participant fears expressed across the theme of emotional response, and apprehension of third parties obtaining their data and/or being located and tracked: *“[…]happy to share all of it, I don’t think that’s really that sensitive, […] as long as it wasn’t linked to my name.” (P124).*

A small number of participants (3 out of 40) mentioned not wanting to share shopping data specifically related to their children: “*I wouldn’t be happy with my children’s data being used” (P101).* However, a small number (2 out of 40) were also happy with this category of data being shared if it was anonymised and could see potential benefits: *“if information is found … children at school should be educated on diet and nutrition” (P119).*

A majority of participants were willing to share their health status, alongside donating their loyalty card data, in order to help researchers investigate specific diseases. Participants appeared to be more comfortable sharing their health status compared to their medical records as a lower level of detail was needed, providing the participant with a higher level of control over what was shared: *“If I could just tick a box, for example, I had a certain condition I’d be okay with that.” (p136).*

A number of participants (17 out of 40) named medical data as more sensitive than other types of personal data, but they still stated they were willing to share this data for research. In contrast to medical data, sharing loyalty card data was frequently viewed as sharing data already in the public domain. Subject to appropriate anonymisation, a large proportion of participants (27 out of 40) were willing to share any data from their shopping regardless of the types of products they bought: *“[…] because if it's anonymised, gathering data about what people are purchasing from a supermarket, I personally wouldn’t find that intrusive.” (P128).*

#### Control and convenience in sharing data

There was no consensus on the control mechanisms and processes for sharing loyalty card data and a few issues were discussed. A majority of participants (24 out of 40) wanted the retailer to take care of donating their data rather than having to donate it directly to the researcher themselves. However, exactly half of participants wanted to be able to have control over which information they wanted to remove from the shopping data before donating it for research. Over half of participants preferred consenting to sharing already collected data, yet almost half would consent to share the data that would be collected about their shopping in the future, too. Despite no consensus on these controls a distinctive theme on convenience emerged from the answers. Participants stated they wanted to donate but were unlikely to do so if the process was not quick and easy. Participants often labelled themselves as lazy and forgetful, or weighed up convenience versus control: *“If it was just all done for me that would be my preference.” (P133).*

#### Transparency, choice and data security

The minority of participants (9 out of 40) did not want to donate to all medical research in general and they preferred to have a choice as to specific application domains. However, there was no consistency as to the types of health research that were inadmissible; only mental health was mentioned by two participants as a particularly sensitive domain. Participants were almost divided equally over whether they attached importance to “choosing” the types of health research their data would be used for: 21 participants did not require a choice at all if it was for the public good, while 18 participants stated that making a choice as to application area was important to their data donation decision. For such participants, transparency about what research they are donating their data for was of value.

Interestingly, even though participants stated they sought an explicit choice, almost half of participants that attached an importance to having such a choice also said there were no health research types they would not donate to. These participants wanted the choice to choose from any domain: *“I’m not overly concerned about where it ends up, or rather, which health sector specifically have it. I would like to maintain the choice [as an option].” (P118).* Corresponding with the theme of transparency, even when participants did not attach an importance to having a choice, it still remained crucial for them to be informed of what the data would be used for.

Finally, transparency was the top requirement given by participants if the public was to be encouraged to trust researchers with their loyalty card data, and was referred to throughout the interview process by participants. Participants wanted it to be clear not only what their loyalty card data was going to be used for, but who would have access to it. Again, participants who desired safeguards were motivated by fears aligned to the emotional response theme. One participant suggested direct interactions would encourage data donation: *“[…]build trust by having conversations with – with people […] make it clear that the university has expectations of its staff and its researchers”. (P140).* While participants referred positively to the high standards universities kept for ethics, working for a good cause or data policies, a quarter of participants still wanted additional reassurances that data would be kept safe and secure.

## Discussion

This study aimed to investigate public attitudes towards sharing loyalty card data for health research with an emphasis on comparing COVID-19 and cancer as the proposed health conditions to be researched, and the safeguards that the public believe should be in place in order for data sharing to occur. The following section synthesises results of the study into recommendations for researchers and the wider policy community seeking to utilise loyalty card data within health research.

### Recommendation 1: Raise awareness of the value of loyalty card data for health research

As envisaged, providing commercially collected data to not-for-profit researchers proved to be a novel concept for many participants interviewed in the study, a finding reflective of previous studies that have explored public opinion on sharing novel types of personal data [25, 26]. Nonetheless, with increased understanding of the usage of this data, participants expressed positive opinions towards donating their loyalty card data for health research. Such affirmations were linked with the way in which loyalty card data were already considered to be in the public domain, and the high level of trust participants placed in universities. Furthermore, willingness to share this data was also linked with prosocial behaviour and the desire to benefit others. Social duty was also found by Skatova and Goulding [24] to be the strongest predictor of whether an individual will choose to donate personal data. However, some participants in the current study expressed potential hesitancy towards sharing data, out of concern for where the data could end up. Likewise, Jones et al. [13] and Skatova et al. [26] found that individuals were concerned that their loyalty card data could be passed on to third parties for profit. Researchers need to ensure that members of the public have an understanding of the ways in which this data can be used for health research, and specifically raise awareness about the value of this type of personal data for improving health outcomes in the general population. Researchers should also consider providing clear examples of how loyalty card data can be used for the specific health condition in question, as in this study it was found that participants could envisage more easily how loyalty card data could be used to research a health condition such as bowel cancer than for COVID-19.

### Recommendation 2: Provide choices for granularity of data sharing

Interviews revealed participants were apprehensive or unwilling to share specific categories within the data. Most commonly, these fears were associated with location data and the way in which this data could be used for tracking and surveillance of their whereabouts. Skatova et al. [26] also found that participants from their study shared concerns about this data being used for ‘live’ tracking if their data revealed they shopped at the same time and place each week. Interestingly, in the current study, participants in the COVID-19 condition frequently raised concerns about data being used to track their movements but were also more likely to share their location data for COVID-19 research in comparison to participants in the cancer research condition. This can be linked with an increased understanding of the importance of location data for COVID-19 research, whereas for cancer research, this link was less obvious. This again raises the importance of providing information on the uses of the categories found within the loyalty card data. In addition, participants may require the choice to opt-out of sharing certain categories if their fears cannot be allayed. The option could also be given to share sensitive categories of data via a more acceptable method such as a demographic survey, which could not infer patterns of movement.

### Recommendation 3: Ensure transparency of data security safeguards

Transparency was most likely to be mentioned as the way in which universities can encourage the public to share their loyalty card data for research, and anonymisation was one of the main areas of concern amongst participants. These findings are consistent with previous research into required safeguards for sharing health data [25]. Universities should therefore ensure that participants are informed about who has access to their data and more generally, that ethical practices and data security policies, in particular data anonymisation or de-identification processes, are accessible. Participants should also be informed of any risks involved in sharing their data so that fears can be allayed. Initiatives such as *Understanding Patient Data* [31] can be used as a reference point. The purpose for which data is being obtained, for the sole use of not-for-profit health research, should also be stressed.

### Recommendation 4: Enable convenient data sharing

Participants were provided with several options in regard to how they would choose to share data. Participants were mostly divided in regard to who should share the data (e.g., retailers vs themselves), who should remove any data they do not want shared, and whether data sharing would take place retrospectively or prospectively. Most participants said they would be happy to donate their data for any form of health research, but half still said they would wish to choose which type of health research their data are used for, which is reflective of previous research [22, 26]. Regardless of preference, participants expressed that convenience was paramount and data sharing should be quick and easy. However, researchers should ensure that convenient data sharing does not jeopardise data security or diminish participant autonomy [32]. One solution, conceived in biomedical research as a way to make consent mechanisms more flexible, is Dynamic Consent [33]. Dynamic Consent uses an electronic portal and allows the researcher to communicate ongoing updates about the study and provides the participant with the option to tailor their consent choices, instead of providing a blanket yes or no consent. Dynamic Consent could be utilised by researchers accessing shopping data in order to facilitate and optimise long-term data sharing.

## Strengths and limitations

This qualitative study has provided an insight into the public’s views on the concept of donating loyalty card data for academic health research. As this was a novel topic for most participants, conducting semi-structured interviews enabled the researchers to introduce the concept of using loyalty card data for health research and address any confusion or unfounded fears. However, recruiting participants via Prolific may have led to sample bias, in particular WEIRD bias (whereby Prolific participants are biased towards Western, Educated, Industrialised, Rich and Democratic Individuals) and rapid responder bias (with responses to participate coming from individuals who are online when the study is launched) [34]. Participants recruited from Prolific, a crowd-sourced research panel, may be more open to data donation than the general public; further, decisions made about donating data by participants were hypothetical in this study. Telephone interviews allowed for convenient interviewing, although may have meant non-verbal cues were lost, and rapport between interviewer and participant was compromised. Comparing responses according to COVID-19 and cancer research also allowed for an analysis of how public acceptability of donating loyalty card data varied according to the type of health condition to be researched. However, participants were randomly allocated into either group and were not asked to consider how their responses would differ according to each health condition, privacy needs might significantly change if the participant had or was at high risk of the disease, and it is unknown whether responses for alternative types of infectious and chronic disease would differ.

The concept of linking health status with loyalty card data was touched upon during the interviews, with most participants expressing willingness to share both forms of data simultaneously for studies using data linkage. Further research exploring the public’s understanding and concerns in regard to sharing these two forms of data for linkage is required. Studies which retrospectively explore participants’ experiences with sharing loyalty card data for research may also add new insights into participants’ views on the process once they have had an opportunity to view their data.

## Conclusions

This study has contributed a set of recommendations, for those researchers seeking to obtain loyalty card data for health research. Whilst participants were largely in favour of donating loyalty card data for academic health research, correct information about the purpose of loyalty card data for research, choice as to what data to share and how it is shared, and appropriate safeguards are all prerequisites for this decision to be reached. Further research into the concept of linking health status with loyalty card data is required. Future research should address the details of data needed in information systems which can link loyalty card data to medical data, and the level of data de-identification at different stages of this process. Studies which retrospectively explore participants’ experiences with sharing loyalty card data for research may also add new insights.

## Supplementary Information

**Additional file 1: Appendix S1.** Interview Guide.

**Additional file 2: Appendix S2. Table S1.** Participant characteristics.

**Additional file 3: Appendix S3. Table S2.** The frequency of participant response types.

## Data Availability

The datasets generated and/or analysed during the current study are not publicly available due to consent not being obtained from participants for this purpose, but are available from the corresponding author on reasonable request. The interview guide can be found in Additional file 1: Appendix S1.

## Abbreviation

ALSPAC: Avon Longitudinal Study of Parents and Children
