# Supplementary material for: Public attitudes towards sharing loyalty card data for academic health research: a qualitative study
Source: BMC Med Ethics. 2022 Jun 7;23:58. doi: 10.1186/s12910-022-00795-8 (PMC9171733; doi:10.1186/s12910-022-00795-8)
Supplement: Supplementary file 1 — Additional file 1: Appendix S1. Interview Guide. [file 12910_2022_795_MOESM1_ESM.docx]

**Appendix S1 Interview Guide**

Icebreaker

We would first like to hear your thoughts on sharing personal data in general for health research.

Q1- Which kinds of data would you class as ‘personal data’?

[Prompts: address, mobile phone data, online shopping history, medical records etc]

Q2- Are there any types of personal data that you would rate as more sensitive than others?

In what ways do you consider these sensitive?

Q3-What are your thoughts on donating personal data for health research purposes? Why?

Understanding

Now, if we could think more about specifically donating loyalty card for health research.

Q4. How would you imagine loyalty card records could be useful for health research in general?

[Prompts: The types on information that are contained within your loyalty card data include where and when you made your purchases, which items you bought, how many items, whether these were discounted, whether they were part of an offer, whether you returned any items. But we wouldn’t be able to see any personal characteristics, such as age, d.o.b or address. However, depending on the data sharing agreement, we may be able to see age or gender, but data would always be anonymised and never reveal your identity.]

Now thinking specifically about donating loyalty card data for research into ovarian or bowel cancer / Covid-19.

Q5-How would you imagine loyalty card records could be useful for research into ovarian or bowel cancer/ Covid-19 ?

[Prompts: by using for instance your supermarket loyalty card data, we can explore whether certain foods are linked with an increased chance in being diagnosed with ovarian or bowel cancer; whether certain over-the-counter medications are linked with an increased chance in suffering better/worse outcomes of Covid-19]

Q6-What benefits do you associate with donating loyalty card records for ovarian or bowel cancer/ Covid-19 research? These could be benefits for yourself and for the general population.

Q7-What negative aspects/ fears do you associate with donating loyalty card records for ovarian or bowel cancer/ Covid-19 research? These could be negative aspects for yourself or for the general population.

Q8- Would you be more or less concerned about donating your loyalty card records than other forms of personal data, such as those we discussed earlier?

Control

In the next part of the interview, we would like to learn more about the level of control you would like to maintain over your loyalty card data, if you were to donate this data for health research

Q9- Which types of (other) health research would you envisage donating your loyalty card data for?

[Prompts: cancer research, mental health research, research into human behaviour e.g. what people were buying during the Covid-19 crisis.]

Q10-Which types of health research, if any, would you not want your loyalty card used for?

Q11-How important is it to you that you would be able to choose the types of health research your data is used for? Why do you attach (or not) this level of importance to having a choice?

Q12-To what extent would you be happy to donate your loyalty card data to a databank and let researchers use it for various different types of health research as and when it was required?

Q13-What spending categories within the loyalty card data would you consider too sensitive or unacceptable to share and why?

[Prompts: medicines, details about purchases related to children]

If we now consider the other types of information that could be revealed if you choose to donate your loyalty card records. This would include: spending category, purchase amount, timestamp, location.

Q14-Which types information within this data are you happy to share and why?

Q15-Which types of information within the data would you not want shared and why?

There would also be different methods of donating your data. For instance: the store provides your loyalty card records directly to the researcher, or you could provide your data to the researcher directly. eg. with Tescos, you can now go onto the website and download your clubcard data onto an excel spreadsheet, which you can directly pass to a researcher. Or you could authorise Tesco to give your data to the researcher.

Q16-What would be your preferred method of donating data and why?

Consider also that you may not want all of the data shared with the researcher.

Q17-Who should remove the data you do not want shared and why (You, store, or researcher)?

There are also different ways of giving your consent for your data to be used. This includes: retrospective, where you provide consent for the researcher to use your loyalty card data from, for example, the last 12 months. Or this could be prospective, so you agree to share your loyalty card records that will be collected in the next 12 months?

Q18-What is your preferred form of consent and why?

You could also have the choice of whether to opt-in or opt-out. So opt-out would mean the store where you hold your loyalty card can transfer your information to researchers automatically at any time unless you tell them you don’t want them to [e.g. new organ donation rules in England]. And opt-in is where the store would have to contact you first before they transfer any information to researchers, and they also could be required to do this every time they transfer your information to a new set of researchers [e.g. like signing up to a newsletter].

Q19-Would you prefer the opt-in or opt-out method of consent and why?

Loyalty card data is much more useful if you also know the health status of that person, such as if they have an illness, and if they do, when they were diagnosed?

Q20- How willing would you be to share your health status alongside donating your loyalty card data, in order to help researchers investigate specific diseases?

Trust

Our final questions are about how much you would trust researchers with your data.

Q21- So to what extent would you trust researchers with your loyalty card data, and why?

[Prompts

- How confident would you be that the university would keep your data securely?

-How confident would you be that the university would use your data for a good cause?]

Q22-What do you think researchers can do to encourage the public to trust them with their loyalty card data?

General

Q23-Do you have any other comments to make in general about this topic?
