# Supplementary material for: Public attitudes towards sharing loyalty card data for academic health research: a qualitative study
Source: BMC Med Ethics. 2022 Jun 7;23:58. doi: 10.1186/s12910-022-00795-8 (PMC9171733; doi:10.1186/s12910-022-00795-8)
Supplement: Supplementary file 2 — Additional file 2: Appendix S2. Table S1. Participant characteristics. [file 12910_2022_795_MOESM2_ESM.docx]

Appendix S2. **Table S1.** Participant characteristics

| **Participant** | **Covid/Cancer Q** | **Age** | **Gender** | **Area** | **Highest education level** | **Previous personal data sharing experience** |
| --- | --- | --- | --- | --- | --- | --- |
| 101 | Covid-19 | 27 | F | South East | GCSEs | Never shared |
| 102 | Covid-19 | 37 | F | East of England | Postgraduate degree | Not sure |
| 103 | Covid-19 | 36 | F | West Midlands | Alevels | Market research |
| 104 | Covid-19 | 19 | M | North West | Alevels | University research Market research |
| 105 | Covid-19 | 42 | F | Yorkshire & the Humber | GCSEs | Not sure |
| 106 | Covid-19 | 20 | M | South East | Alevels | Not sure |
| 107 | Covid-19 | 20 | F | South East | Alevels | Never shared |
| 108 | Covid-19 | 18 | F | South West | Alevels | Never shared |
| 109 | Covid-19 | 42 | F | South East | Undergraduate degree | Never shared |
| 110 | Covid-19 | Prefer not to say | F | West Midlands | Alevels | Not sure |
| 111 | Covid-19 | 28 | M | South East | Undergraduate degree | Charitable research |
| 112 | Covid-19 | 44 | M | West Midlands | Postgraduate degree | Market research |
| 113 | Covid-19 | 43 | M | Scotland | Undergraduate degree | Not sure |
| 114 | Covid-19 | 27 | F | East Midlands | Postgraduate degree | University research Market research |
| 115 | Covid-19 | 61 | M | Scotland | Postgraduate degree | University research  Market research |
| 116 | Bowel Cancer | 40 | M | Scotland | GCSEs | Never shared |
| 117 | Bowel Cancer | 19 | M | South East | Alevels | Not sure |
| 118 | Bowel Cancer | 18 | M | North West | Alevels | Never shared |
| 119 | Ovarian / Bowel Cancer | 40 | F | East of England | Alevels | University research Market research |
| 120 | Bowel Cancer | 19 | M | South East | GCSEs | Market research |
| 121 | Bowel Cancer | 48 | M | Yorkshire & the Humber | Postgraduate degree | Not sure |
| 122 | Ovarian / Bowel Cancer | 27 | F | South East | Undergraduate degree | Not sure |
| 123 | Bowel Cancer | 54 | M | South West | GCSEs | University research  Market research  Charitable research |
| 124 | Ovarian / Bowel Cancer | 29 | F | South East | Undergraduate degree | University research  Market research  Charitable research |
| 125 | Ovarian / Bowel Cancer | 36 | F | South West | Undergraduate degree | Market research |
| 126 | Covid-19 | Prefer not to say | M | Yorkshire & the Humber | Prefer not to say | Not sure |
| 127 | Covid-19 | 30 | F | East of England | Undergraduate degree | Not sure |
| 128 | Covid-19 | 48 | F | East of England | Prefer not to say | Never shared |
| 129 | Covid-19 | 26 | M | North West | Postgraduate degree | Never shared |
| 130 | Covid-19 | 24 | F | West Midlands | Undergraduate degree | Never shared |
| 131 | Bowel Cancer | 39 | M | South East | Undergraduate degree | Charitable research  Market research |
| 132 | Ovarian / Bowel Cancer | 27 | F | Yorkshire & the Humber | Undergraduate degree | Market research |
| 133 | Ovarian / Bowel Cancer | 27 | F | Scotland | Undergraduate degree | University research  Market research |
| 134 | Ovarian / Bowel Cancer | 29 | F | South East | Undergraduate degree | Never shared |
| 135 | Ovarian / Bowel Cancer | 64 | F | South West | Postgraduate degree | Not sure |
| 136 | Ovarian / Bowel Cancer | 24 | F | South East | Undergraduate degree | Never shared |
| 137 | Bowel Cancer | 40 | M | Yorkshire & the Humber | Undergraduate degree | Market research |
| 138 | Ovarian / Bowel Cancer | 28 | F | Scotland | Postgraduate degree | University research |
| 139 | Bowel Cancer | 24 | M | South East | Undergraduate degree | University research  Market research  Charitable research |
| 140 | Bowel Cancer | 59 | M | East of England | Postgraduate degree | Not sure |
