# Supplementary material for: Public attitudes towards sharing loyalty card data for academic health research: a qualitative study
Source: BMC Med Ethics. 2022 Jun 7;23:58. doi: 10.1186/s12910-022-00795-8 (PMC9171733; doi:10.1186/s12910-022-00795-8)
Supplement: Supplementary file 3 — Additional file 3: Appendix S3. Table S2. The frequency of participant response types. [file 12910_2022_795_MOESM3_ESM.docx]

Appendix S3. **Table S2.** The frequency of participant response types

|  | **Question** | **Answer** | **Frequency (N=40)** |
| --- | --- | --- | --- |
| 1 | Are there any types of personal data that you would rate as more sensitive than others? | Health/Medical Data | 16 |
|  |  | Address | 9 |
|  |  | Finance/Banking Data | 8 |
|  |  | D.O.B | 5 |
|  |  | Name | 4 |
|  |  | National Insurance No. | 3 |
|  |  | Age | 3 |
|  |  | Personally identifiable data | 3 |
|  |  | Other | 20 |
| 2 | What are your thoughts on donating personal data for health research purposes? | Positive response | 27 |
|  |  | Ok with it | 7 |
|  |  | Depends on circumstances | 3 |
|  |  | Negative response | 2 |
| 3 | Would you be more or less concerned about donating your loyalty card records than other forms of personal data? | Would be less concerned | 37 |
|  |  | Would be more concerned | 1 |
|  |  | Not sure | 2 |
| 4 | Which types of health research, if any, would you not want your loyalty card used for? | I would not exclude any | 31 |
|  |  | Mental health | 2 |
|  |  | Other | 7 |
| 5 | How important is it to you that you would be able to choose the types of health research your data is used for? | Choice not important | 21 |
|  |  | Choice important | 18 |
| 6 | To what extent would you be happy to donate your loyalty card data to a databank and let researchers use it for various different types of health research as and when it was required? | Happy to donate | 30 |
|  |  | Fine/Fairly happy | 3 |
|  |  | Not opposed | 1 |
|  |  | It depends | 2 |
|  |  | No, would not donate | 4 |
| 7 | What spending categories within the loyalty card data would you consider too sensitive or unacceptable to share and why? | Would share all categories | 24 |
|  |  | Medicines | 6 |
|  |  | Categories related to children | 4 |
|  |  | Sexual health products | 2 |
| 8 | Which types of information within this data are you happy to share and why? Spending category, purchase amount, timestamp, location. | Would donate all types | 28 |
|  |  | Would not donate location | 9 |
|  |  | Would not donate timestamp | 5 |
| 9 | What would be your preferred method of donating data and why?5 | The retailer donates the data | 24 |
|  |  | The participant donates data | 13 |
|  |  | Depends / No preference | 3 |
| 10 | Who should remove the data you do not want shared? | Themselves | 20 |
|  |  | The retailer | 12 |
|  |  | The researchers | 4 |
|  |  | Themselves & retailer | 4 |
|  |  | Themselves, the retailer & researchers | 1 |
| 11 | What is your preferred form of consent and why? | Retrospective | 21 |
|  |  | Prospective | 7 |
|  |  | Both | 12 |
| 12 | Would you prefer the opt-in or opt-out method of consent and why? | Opt-in | 23 |
|  |  | Opt-out | 16 |
|  |  | Either | 1 |
| 13 | How willing would you be to share your health status alongside donating your loyalty card data, in order to help researchers investigate specific diseases? | Would share | 33 |
|  |  | Would not share | 6 |
|  |  | It depends | 1 |
| 14 | To what extent would you trust researchers with your loyalty card data? | Would trust | 31 |
|  |  | It depends | 4 |
|  |  | Somewhat | 2 |
|  |  | Not sure | 1 |
|  |  | No | 1 |
| 15 | What do you think researchers can do to encourage the public to trust them with their loyalty card data? | Transparency | 20 |
|  |  | Raise awareness | 6 |
|  |  | Data security | 6 |
|  |  | Show benefits | 5 |
|  |  | Anonymised | 3 |
|  |  | Share outcomes | 3 |
|  |  | Be honest | 2 |
|  |  | Make it accessible | 2 |
|  |  | Other | 11 |
